# An unusual case of colonic duplication cyst in an adult with dysplasia

**DOI:** 10.1093/jscr/rjad039

**Published:** 2023-02-21

**Authors:** Aswin Shanmugalingam, Hayley Duxbury, Joseph Do Woong Choi, Charlotte Kwik, Chow Heok P’Ng, Lauren Kim, Nimalan Pathma-Nathan

**Affiliations:** Department of Colorectal Surgery, Westmead Hospital, Corner of Hawkesbury and Darcy Roads, Westmead, NSW 2145, Australia; Department of Tissue Pathology and Diagnostic Oncology, ICPMR, Westmead Hospital, Corner of Hawkesbury and Darcy Roads, Westmead, NSW 2145, Australia; Department of Colorectal Surgery, Westmead Hospital, Corner of Hawkesbury and Darcy Roads, Westmead, NSW 2145, Australia; Department of Colorectal Surgery, Westmead Hospital, Corner of Hawkesbury and Darcy Roads, Westmead, NSW 2145, Australia; Department of Tissue Pathology and Diagnostic Oncology, ICPMR, Westmead Hospital, Corner of Hawkesbury and Darcy Roads, Westmead, NSW 2145, Australia; Department of Tissue Pathology and Diagnostic Oncology, ICPMR, Westmead Hospital, Corner of Hawkesbury and Darcy Roads, Westmead, NSW 2145, Australia; Department of Colorectal Surgery, Westmead Hospital, Corner of Hawkesbury and Darcy Roads, Westmead, NSW 2145, Australia

## Abstract

Duplication cysts are rare benign congenital malformations typically identified in children by the age of 2 years. We report a rare case of colonic duplication cyst with dysplasia in an adult. A 32-year-old male was diagnosed with non-specific abdominal symptoms. Abdominopelvic computed tomography scan demonstrated a submucosal cystic lesion in the right colon. He underwent laparoscopic right hemicolectomy. Histopathology showed colonic duplication cyst with low grade dysplasia. He is due for a surveillance colonoscopy in 3 years. Duplication cyst in an adult colon with dysplasia is extremely rare. They are usually present in the terminal ileum. They have non-specific abdominal symptoms or can be asymptomatic. They are often identified incidentally or intraoperatively. Imaging may demonstrate a cystic lesion. Histopathology is required for definitive diagnosis. There are no guidelines or consensus on managing duplication cysts in adults. We recommend an oncological resection of the involved colon. Surveillance with routine colonoscopy is advisable.

## INTRODUCTION

Gastrointestinal duplication cysts are rare congenital malformations of the gastrointestinal tract that form between the fourth and eighth weeks of development [1]. This malformation usually occurs in the ileum (60%) followed by the jejunum and duodenum [1]. They are typically considered a benign condition that often present by the age of 2 [1–3]. The authors present an interesting case of a right colonic duplication cyst with low grade dysplasia in an adult.

## CASE REPORT

A 32-year-old male was admitted to the Emergency Department with a one-week history of intermittent right lower quadrant (RLQ) colicky abdominal pain. He was not clinically obstructed, and denied associated symptoms including altered bowel habit, per rectal bleeding and unintentional weight loss. He had no relevant background medical or surgical history, and there was no family history of colorectal cancer. He migrated to Australia 10 years ago from Sri Lanka, and had no recent contact with livestock. His vital signs were within normal limits. The abdominal examination elicited mild RLQ tenderness without peritonism, palpable mases or organomegaly.

**Figure 1:**
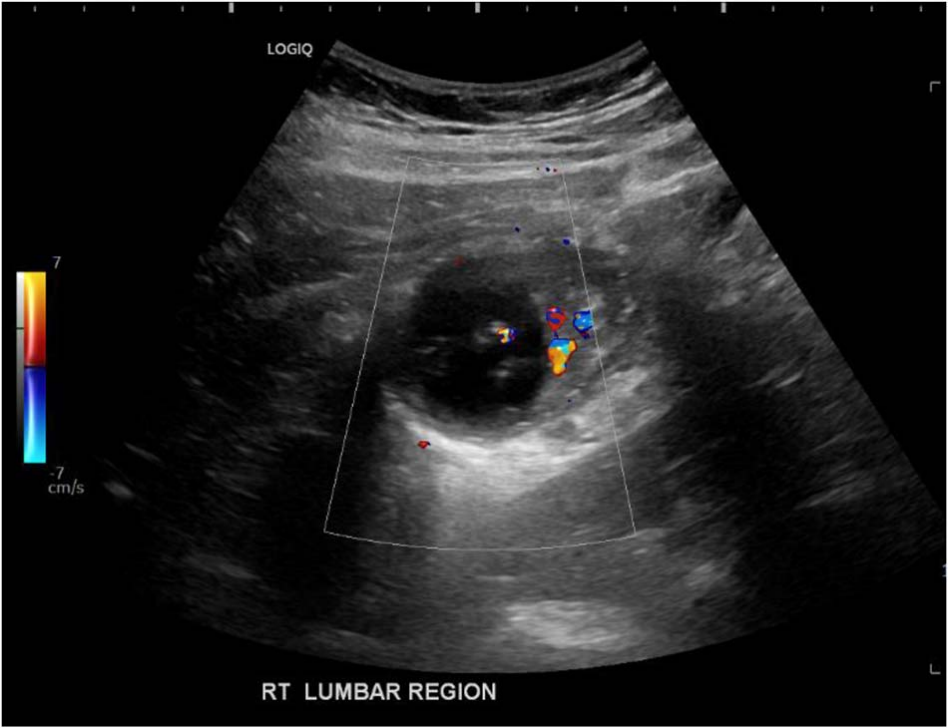
Ultrasound image demonstrating a cystic lesion with internal calcification and no hypervascularity.

**Figure 2:**
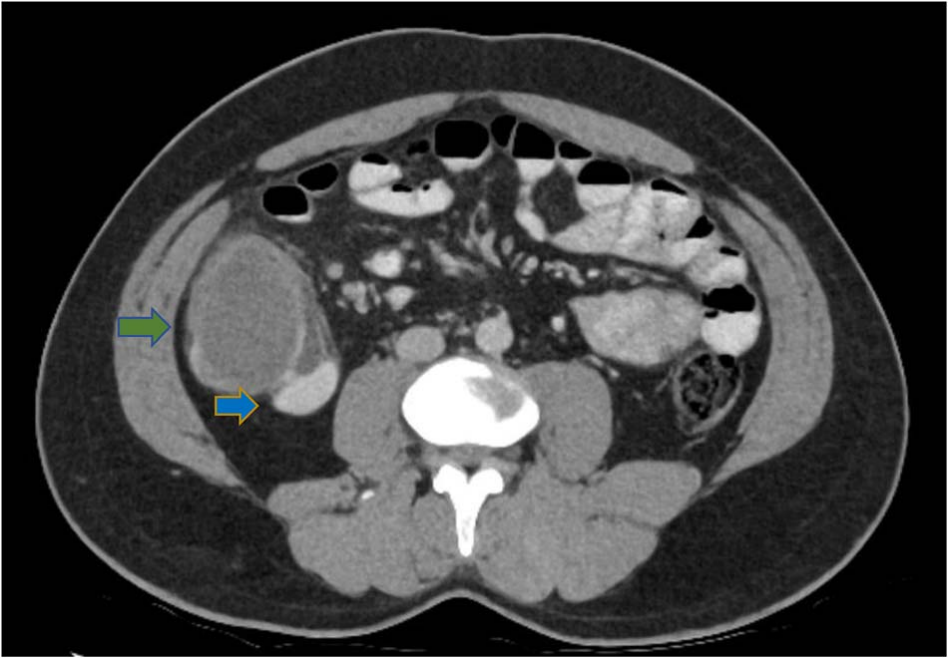
Axial CT abdomen demonstrating a cystic submucosal lesion at the right colon (green arrow). There is involvement of the colonic lumen but no obstruction with oral contrasting passing through the lumen (blue arrow).

His white cell count was 12.7 × 10^9^/L (reference range 3.7–9.5 × 10^9^/L) and c-reactive protein was 39 mg/L (reference range ≤ 4 mg/L). Hydatid and entamoeba serologies were negative. Carcinoembryonic antigen was elevated to 11.4 ug/L (reference range ≤ 3 ug/L). Abdominal ultrasound showed an uncharacterizable RLQ cystic structure with calcification (Fig. 1). Subsequent computed tomography (CT) scan demonstrated a 56 mm × 43 mm × 58 mm ovoid ascending colon mass with central low density and rim enhancement (Fig. 2). Ileocolic lymphadenopathy up to 7.5 mm in diameter was noted with no evidence of distant metastatic disease.

**Figure 3:**
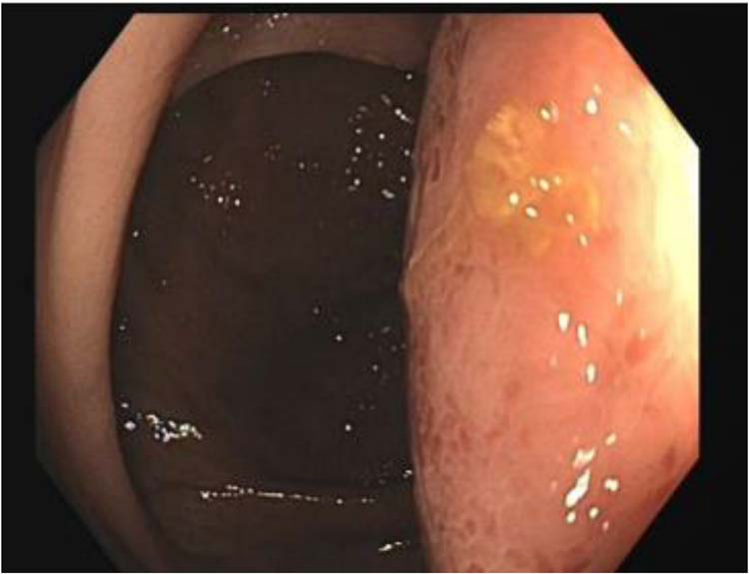
Colonoscopy view of the colonic duplication cyst.

A number of differential diagnoses were considered including neoplastic and infectious pathologies such as latent TB, hydatid or amoebic cyst. A subsequent colonoscopy showed a submucosal semi-pedunculated lesion in the ascending colon measuring greater than 50 mm (Fig. 3). Biopsies were histologically nonspecific. As the patient was symptomatic of this lesion, and moreover as a malignant process could not be excluded, he proceeded with a surgical resection.

Intraoperatively a palpable, freely mobile ascending colon mass was found. There was no macroscopic evidence of peritoneal disease. A laparoscopic right hemicolectomy with ileocolic anastomosis was performed. He made an uneventful recovery and was discharged at post-operative day 5.

**Figure 4:**
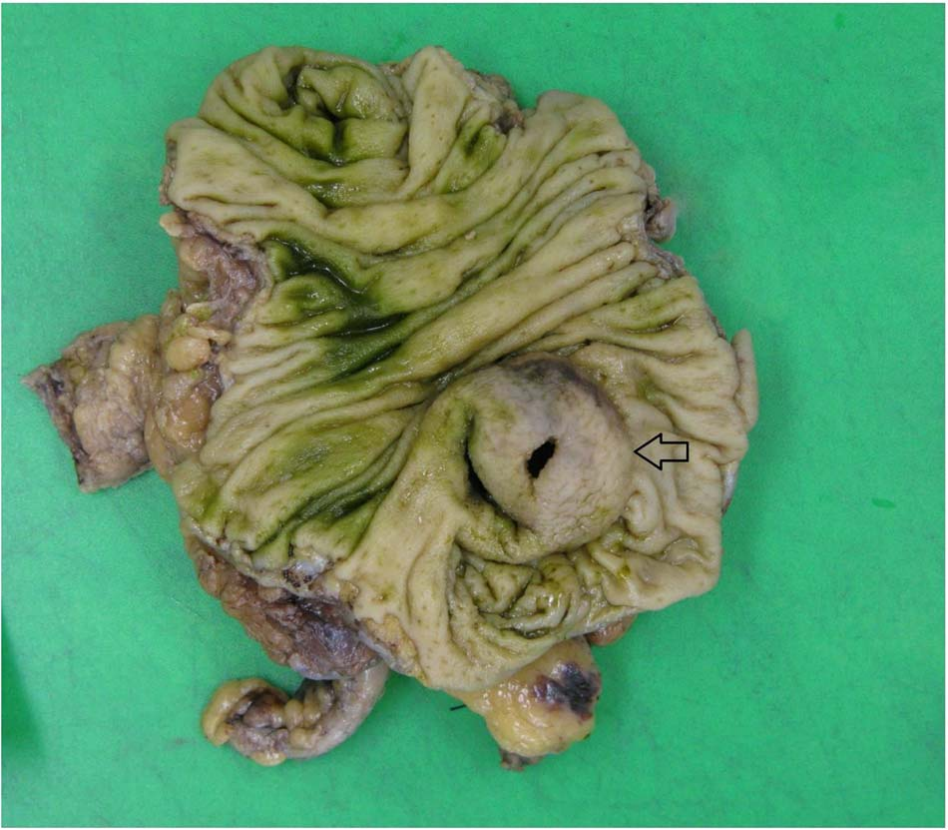
Macroscopic view of specimen post-fixation, with duplication cyst visible at lower right within caecum (arrow).

**Figure 5:**
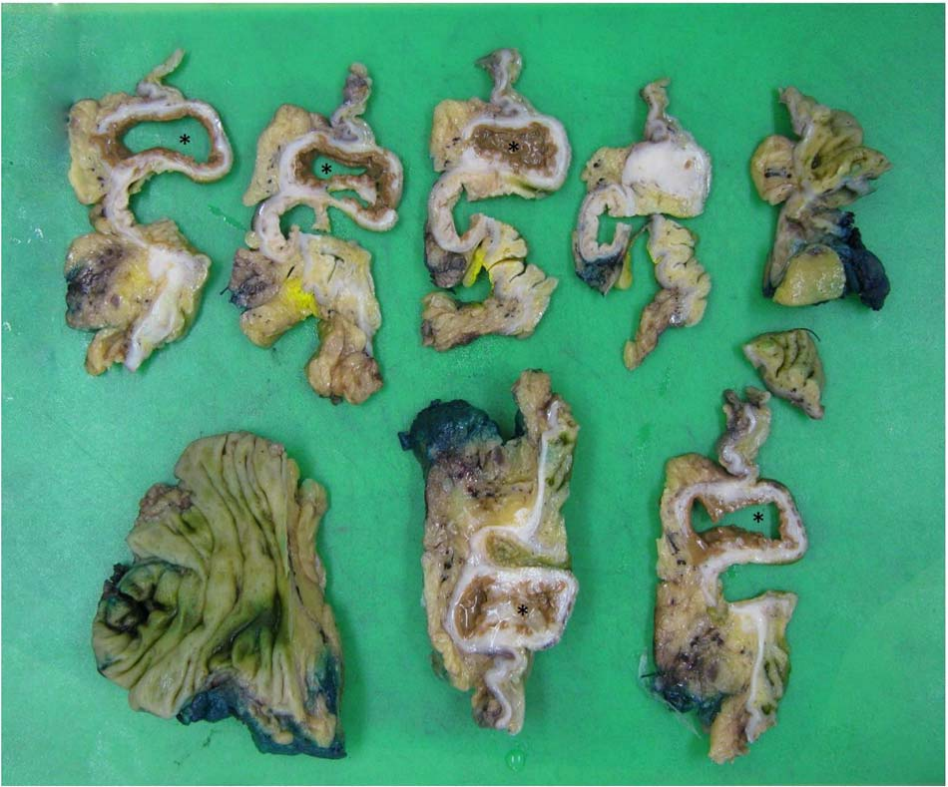
Dissection of specimen; duplication cyst (asterisk) with adjacent bowel wall visible.

**Figure 6:**
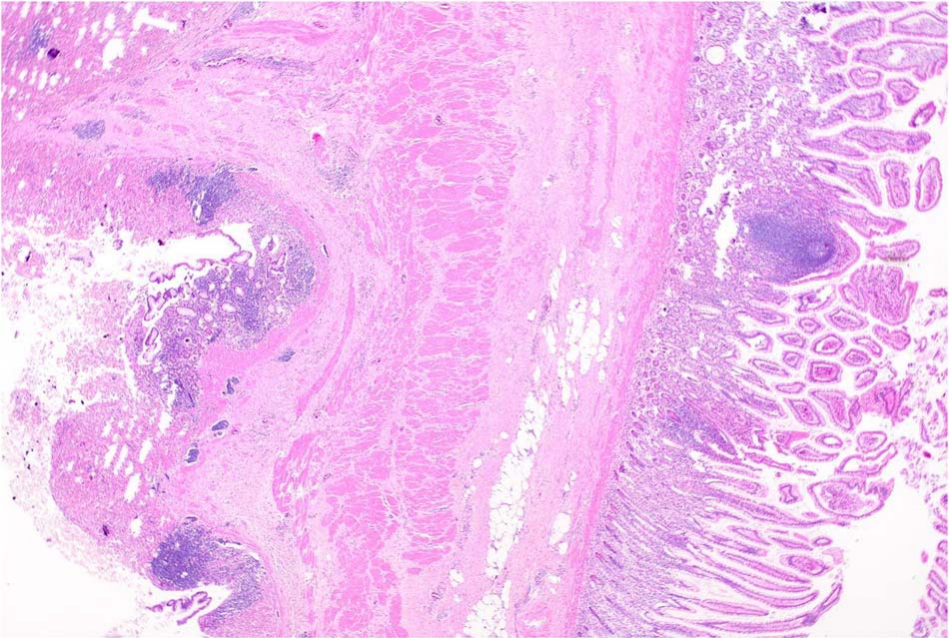
Remnant of normal colonic epithelium (left) within the duplication cyst that shares common muscularis propria with the terminal ileum (right), low power.

**Figure 7:**
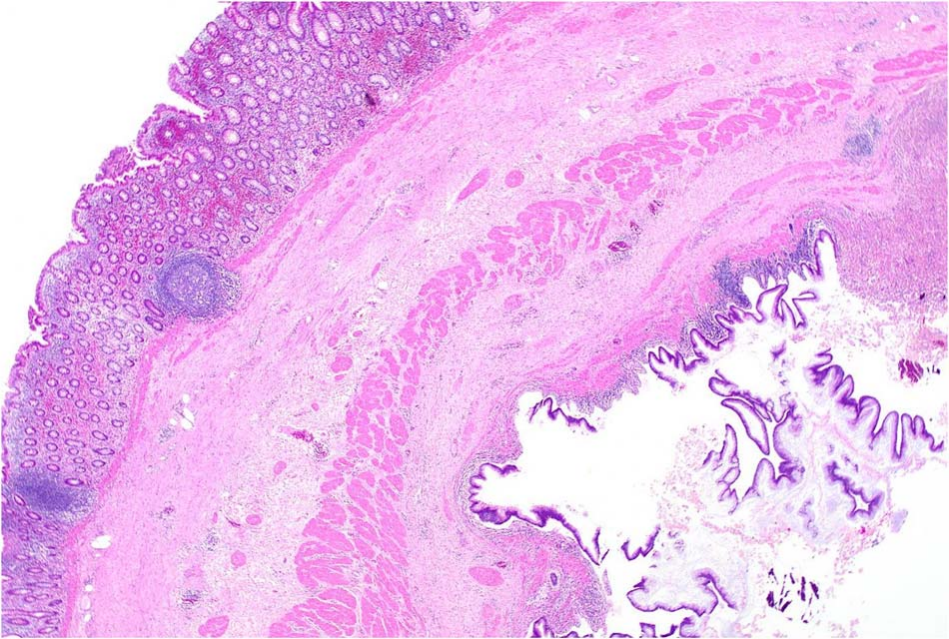
Area of dysplastic mucosa (right) within the duplication cyst that shares a common wall with the right colon (left), low power.

**Figure 8:**
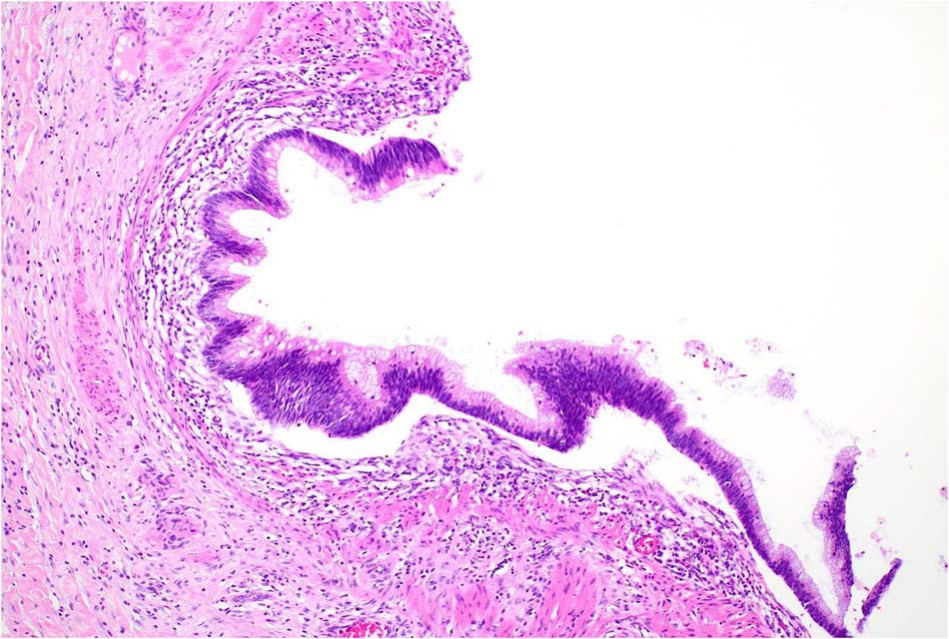
Low grade dysplasia in duplication cyst epithelium, high power.

Histopathology macroscopically identified a 42-mm cystic caecal mass adjacent to the ileocaecal valve (Figs 4–5). Microscopic examination revealed a duplication cyst involving the ileal and colonic tissue comprised of a complete duplication of the colonic wall including mucosa, submucosa and muscularis propria, which was shared with the involved colon (Fig. 6). There was no mucosal connection to adjacent normal bowel. There was some ulceration with inflammatory changes in the overlying mucosa suggestive of prior cyst perforation and areas of attenuated villiform mucinous epithelium with features of low grade dysplasia (Figs 7-8). Special stains for organisms (Periodic Acid-Schiff (PAS), Fredericamycin A (FMA), Ziehl-Neelsen (ZN) and modified ZN) were negative. There were no granulomas, heterotopic mucosa or evidence of malignancy. Twenty-nine lymph nodes were identified with no evidence of malignancy.

Given these findings, he was recommended to undergo surveillance colonoscopy after 3 years.

## DISCUSSION

Duplication cysts are congenital malformations that can form anywhere along the gastrointestinal tract at the mesenteric side [3]. More commonly affecting males, the overall estimated incidence is 1:4500 births [4–6]. Large bowel duplication cysts are rare, comprising only 6.8% of all duplication cysts [7, 8].

By definition, they are comprised of a complete duplication of all layers of gastrointestinal tissue, including the mucosa, submucosa and a smooth muscle layer [5, 6, 8]. They often share the smooth muscle layer and the blood supply with the adjacent normal bowel, except for a rare variant of isolated duplication cysts [9, 10]. The mucosa may resemble adjacent tissue, or be heterotopic gastric, pancreatic or another type [8, 10, 11]. In this case, the colonic wall was completely replicated, with additional areas of epithelial ulceration, inflammation and low grade dysplasia. The aetiology and pathogenesis of duplication are not yet fully elucidated; theories include the failure of intestinal recanalization during embryological development, partial twinning, the split notochord theory, persistence of foetal diverticulum and intrauterine vascular malformation [4–6, 9, 12].

Colonic duplication cysts can be asymptomatic or present with vague abdominal symptoms as seen in this case. Large cysts may be present with obstructive symptoms. Gastrointestinal bleeding may also be seen if heterotopic gastric mucosa is present, causing ulceration into adjacent vessels or organs [2, 5, 6, 13]. There are also reports of duplication cysts diagnosed with intussusception, volvulus and perforation [12].

Diagnosis of colonic duplication cyst is reliant on radiology, intra-operative findings and histopathology. The typical finding on ultrasonography (US) is a cystic lesion with hypoechoic outer rim representing the smooth muscle layer [3, 7]. Endoscopic US may additionally demonstrate submucosa and mucosa as inner hyperechoic layers [3, 7]. CT typically demonstrates a fluid filled cystic lesion or a thin walled tubular structure. As in this case, a CT scan with oral contrast may be utilized to evaluate for bowel obstruction.

Malignant changes within duplication cysts are extremely rare; however, there have been a few reports of malignancy detected within the duplication cysts, most commonly in adults [1, 3, 4, 14]. These may be present at an advanced stage in the young adult population, though, fortunately, our patient only had low grade dysplasia [12]. The recommended treatment in both symptomatic and asymptomatic patients is an oncological surgical resection of the involved colon [3, 6, 12]. It is difficult to achieve isolated cyst excision as they share tissue and vasculature with the colon, although it may be possible if there is an independent blood supply to the cyst [15]. The specimen must be histologically examined for dysplasia, and inclusion of lymph nodes in the resection is essential for staging if there are malignant changes within the cyst [1]. Alternatively, endoscopic resection of the cyst roof with a snare device has also been reported [4]. However, this requires closer monitoring with 6-month surveillance colonoscopy, as malignant changes cannot be excluded even with multiple biopsies of the cystic epithelium.

Standardized guidelines for monitoring patients with dysplasia or malignant changes within duplication cysts are lacking. This may need to be determined on a case-by-case basis guided by histopathology. With features of low grade dysplasia rather than overt malignancy, the decision in this case was for a 3-year follow-up with surveillance colonoscopy.

## CONSENT

Written and verbal informed consent was obtained from the patient to publish this case report and accompanying images.

## CONFLICT OF INTEREST STATEMENT

None declared.

## FUNDING

None.

## DATA AVAILABILITY

The authors are able to provide data upon request.
