# Correction to: An unusual case of colonic duplication cyst in an adult with dysplasia

**DOI:** 10.1093/jscr/rjad150

**Published:** 2023-03-15

This is a correction to: Aswin Shanmugalingam, Hayley Duxbury, Joseph Do Woong Choi, Charlotte Kwik, Chow Heok P'Ng, Lauren Kim, Nimalan Pathma-Nathan, An unusual case of colonic duplication cyst in an adult with dysplasia, *Journal of Surgical Case Reports*, Volume 2023, Issue 2, February 2023, rjad039, https://doi.org/10.1093/jscr/rjad039

In the originally published version of this manuscript, reference #13 was missing: “Cavallaro G, Arena R, D'Ermo G, *et al.* Cystic duplication of transverse colon: an unusual case of abdominal pain and bowel obstruction. *G Chir* 2010;**31**:236–238.”

The reference “Brian PH, Hyrcza MH, Ramsay J, Tse F. Adenocarcinoma arising from a gastric duplication cyst. *ACG Case Rep J* 2018;**5**:e42” should be labelled as reference #14.

The reference “Srivastava P, Gangopadhyay AN, Kumar V, Upadhyaya VD, Sharma SP, Jaiman R, *et al.* Noncommunicating isolated enteric duplication cyst in childhood. *J Paediatr Surg* 2009;**44**:E9–E10” should be labelled as reference #15.

The sentence “Gastrointestinal bleeding may also be seen if heterotopic gastric mucosa is present, causing ulceration into adjacent vessels or organs [2, 5, 6]” should reference “[2, 5, 6, 13]” instead,

The sentence “Malignant changes within duplication cysts are extremely rare; however, there have been a few reports of malignancy detected within the duplication cysts, most commonly in adults [1, 3, 4, 13]” should reference “[1, 3, 4, 14]” instead,

The sentence “It is difficult to achieve isolated cyst excision as they share tissue and vasculature with the colon, although it may be possible if there is an independent blood supply to the cyst [14]” should reference “[15]” instead.

These errors have now been corrected.

